# Descriptions of two new species of the genus *Laius* Guérin-Méneville, 1838 (Coleoptera, Melyridae, Malachiinae) from China

**DOI:** 10.3897/zookeys.1283.184622

**Published:** 2026-06-26

**Authors:** Zhenhua Liu, Fuwen Qiu, Zhiqiang Li

**Affiliations:** 1 Guangdong Key Laboratory of Animal Conservation and Resource Utilization, Institute of Zoology, Guangdong Academy of Sciences, Guangzhou 510260, China Third Institute of Oceanography, Ministry of Natural Resources Xiamen China; 2 Third Institute of Oceanography, Ministry of Natural Resources, Xiamen 361005, China Guangdong Key Laboratory of Animal Conservation and Resource Utilization, Institute of Zoology, Guangdong Academy of Sciences Guangzhou China

**Keywords:** Apalochrini, Beetle, seashore, taxonomy, Xiamen

## Abstract

The genus *Laius* (Melyridae, Malachiinae) inhabits seashore rocks and currently includes five species from China, two of which require transfer to other genera based on their original descriptions. Two new sympatric species from Fujian, *Laius simingensis***sp. nov**. and *Laius xiamenensis***sp. nov**., are described. A diagnostic key, based on males, to the valid Chinese species of *Laius* is provided. The distribution of *Laius flavicornis* (Fabricius, 1801) in China is discussed.

## Introduction

The genus *Laius* Guérin-Méneville, 1838, belonging to the tribe Apalochrini of malachiine Melyridae, inhabits seashore rocks from East Africa to South-East Asia, Australia, and some Pacific islands (Yoshitomi 2014; Plonski and Háva 2020). Historically, this genus encompassed numerous species characterized by enlarged, modified basal antennomeres in males. However, many of these species were subsequently transferred to several other genera based on distinct male-specific characters (Wittmer 1952, 1997; Evers 1994; Yoshitomi and Lee 2010; Plonski 2013, 2014a, 2014b, 2016; Liu et al. 2015, 2017, 2020). In China, 12 species were previously included in *Laius* in the Catalogue of Palearctic Coleoptera (Mayor 2007), but most have since been transferred to the genus *Intybia* Pascoe, 1866 (Wittmer 1997; Yoshitomi and Lee 2010; Plonski 2013, 2014a, 2016), and two additional species were described from Taiwan (Yoshitomi and Lee 2010). Consequently, five species of *Laius* are currently recognised in China. However, *L. pici* Miwa, 1931 has been noted for its similarity to *Intybia* (Yoshitomi and Lee 2010), and *L. theresae* Pic, 1944 is also expected to be transferred to related genera based on its original description (Pic 1944; Mayor 2007). Thus, only three species of *Laius* recorded in China are considered reliably confirmed: *L. lutaoensis* Yoshitomi & Lee, 2010 and *L. taiwanus* Yoshitomi & Lee, 2010 from Taiwan, and *L. flavicornis* (Fabricius, 1801), which was recorded from China by Champion (1921) without a specific locality.

No records of *Laius* had been reported from mainland China since 1921 (Champion 1921), and no additional specimen from mainland China were examined until field observations were conducted on seashore rocks in Xiamen in June 2025. Consequently, two new species of *Laius* are described here based on a large series of newly collected specimens.

## Materials and methods

Specimens studied are deposited in the following institutions: Institute of Zoology, Guangdong Academy of Sciences, Guangzhou, China (**IZGAS**); Australian National Insect Collection, Canberra, Australia (**ANIC**).

The morphological terms used in this paper follow Lawrence and Ślipiński (2013) and Yoshitomi (2014). Measurements were made as follows: body length—from the apical edge of the clypeus to the apex of the elytra; pronotal length—median line from the anterior margin to the posterior one; pronotal width—maximum width of the pronotum; elytral length—from the base of the elytra and scutellum to the elytral apex along the suture; elytral width—maximum width across the elytra.

Specimens’ abdomens for dissections were immersed to 10% KOH solution for about 12 h at room temperature, then transferred to open cavity slides for dissection under a Leica SAPO stereoscope. Dry specimens were mounted on cards with white emulsion glue; genitalia and terminal abdominal segments are preserved in genitalia vials with glycerol.

Layered images were captured using a Canon 7D DSLR camera, Canon MPE 65 mm macro lens or Mitutoyo 5×/10× objective lens on an adapter, with Helicon Remote v. 3.9.10 and WeMacro control software. the camera was mounted on the focus stacking rail with a dual-headed flash. The images of the male genitalia were stacked in Helcion Focus v. 8.1.1 and edited in Photoshop CC 2022.

## Taxonomy

### Melyridae Leach, 1815

**Malachiinae Fleming, 1821**

#### Laius

Taxon classificationAnimaliaColeopteraMelyridae

Guérin-Méneville, 1838

22792A90-AEFF-511D-90DE-447F3D4BD23D

##### Type species.

*Laius cyaneus* Guérin-Méneville, 1838.

##### Chinese common name.

滨囊花萤属.

##### Diagnosis.

This genus can be distinguished from other Apalochrini by the following combination of characters: body black with metallic bluish or greenish lustre; antennomeres 1 and 3 dilated and modified in male, remaining segments filiform; protibiae in males enlarged and curved at the basal third, with inner surfaces concave near base; protarsi 5-segmented in both sexes, without toothed extension.

##### Remarks.

Apart from the morphological characters, its special habitat is also unique in Apalochrini, with both larvae and adults living on seashore rocks, feeding on animal proteins of other small invertebrates, like oysters and barnacles.

#### Laius simingensis sp. nov.

Taxon classificationAnimaliaColeopteraMelyridae

4143DD66-BE02-55F3-8452-DCDADCAF5339

https://zoobank.org/FD045B81-1E3D-465E-B1CC-5914B5C667BF

**Figure 1.**
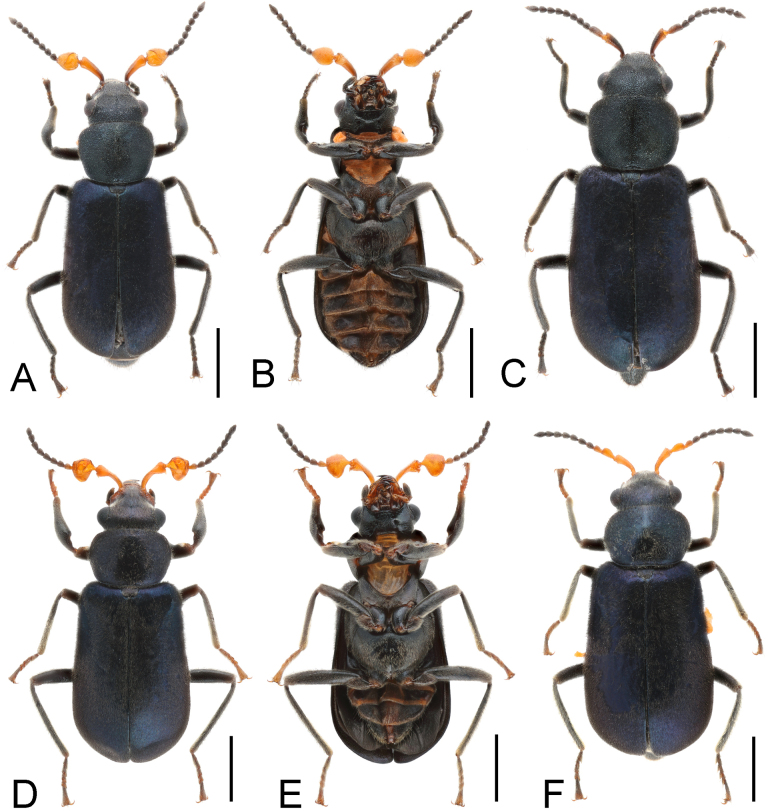
Habitus of *Laius* species. **A–C**. *L. simingensis* sp. nov.; **D–F**. *L. xiamenensis* sp. nov.; **A, D**. Dorsal view, male, holotype; **B, E**. Ventral view, male, holotype; **C, F**. Dorsal view, female, paratype. Scale bars: 1 mm.

**Figure 2.**
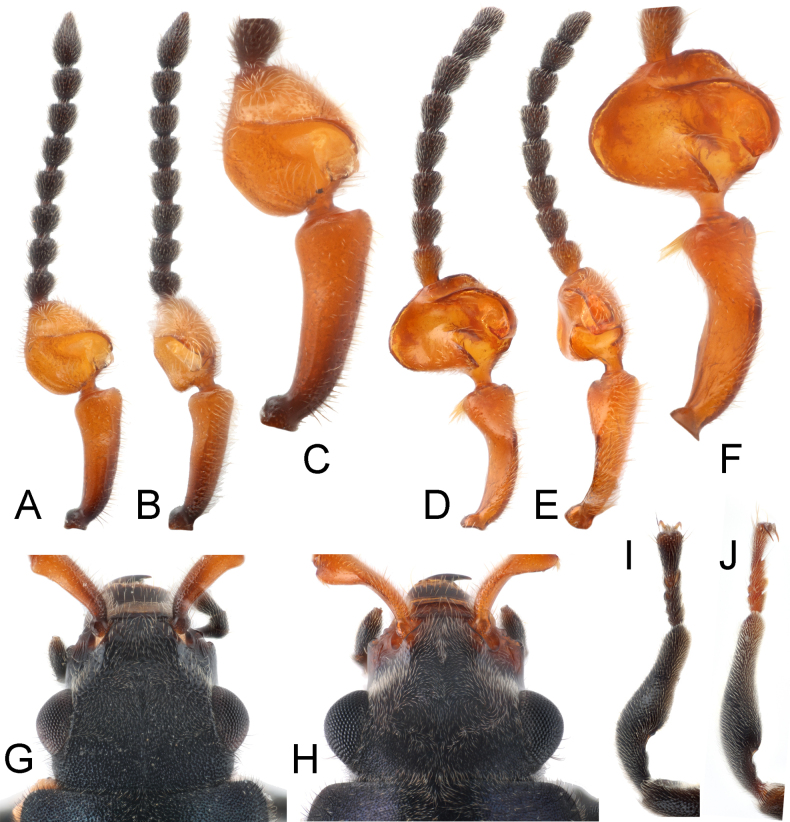
Male characters of *Laius* species. **A–C, G, I**. *L. simingensis* sp. nov.; **D–F, H, J**. *L. xiamenensis* sp. nov.; **A, D**. Antennae, dorsal view; **B, E**. Antennae, lateral view; **C, F**. Basal antennomeres, dorsal view; **G, H**. Head, dorsal view; **I, J**. Protibia and tarsus, dorsal view.

**Figure 3.**
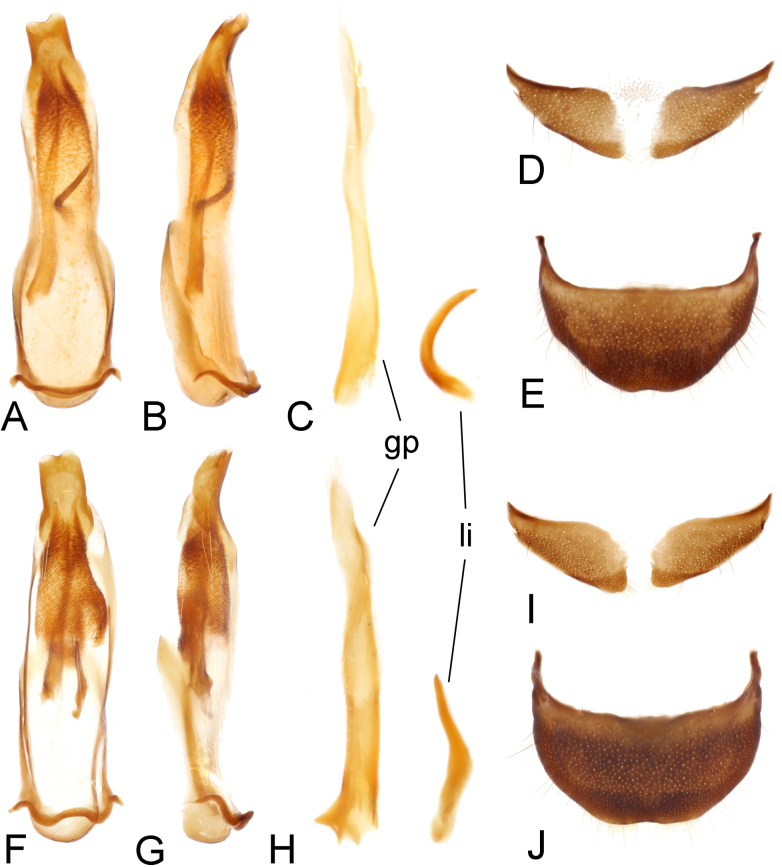
Aedeagus and terminal abdominal segments of *Laius* species. **A–E**. *L. simingensis* sp. nov.; **F–J**. *L. xiamenensis* sp. nov.; **A, F**. Penis, dorsal view; **B, G**. Penis, lateral view; **C, H**. Sclerites in endophallus; **D, I**. Sternite VIII; **E, J**. Tergite VIII. Abbreviations: gp – gonoporal piece, li – ligula.

**Figure 4.**
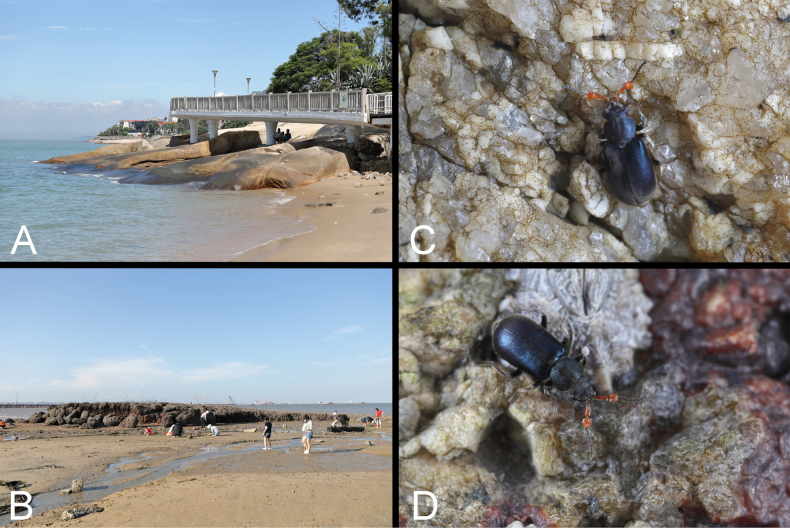
Ecological photos. **A**. Zhenzhuwan beach, Xiamen; **B**. Guanyinshan beach, Xiamen; **C**. *Laius xiamenensis* sp. nov.; **D**. *Laius simingensis* sp. nov.

Figs 1A–C, 2A–C, 2G, 2I, 3A–E, 4D

##### Chinese common name.

思明滨囊花萤.

##### Type material.

***Holotype***: • male, “CHINA. Fujian, Xiamen, Siming District, Guanyinshan beach, E118.197125 N24.492729, 2025.vi.21–22, leg. Zhenhua Liu” (IZGAS). ***Paratypes***: • 8 males, 18 females, same data as the holotype (IZGAS); • 10 males, 8 females, “CHINA. Fujian, Xiamen, Siming District, Zhenzhuwan beach, E118.112624 N 24.429981, 2025.vi.21–22, leg. Zhenhua Liu” (IZGAS); • 1 male, 2 females, “CHINA. Fujian, Quanzhou, Jinmen County, E118.338654 N24.404996, 2025.vi.21, leg. Yuchen Zheng” (IZGAS).

##### Diagnosis.

This species belongs to species group 3 of Yoshitomi (2014), with a long gonoporal piece and a short and curved ligula (Fig. 3C). It mostly resembles *L. miyamotoi* in colouration and shape of basal antennomeres (Nakane 1955), with the inner side of scape darkish, antennomere 3 with a large concavity at its base and a small, rounded concavity near its apex. However, *L. simingensis* can be separated from *L. miyamotoi* by the longer ligula, with the gonoporal piece about 3.3 times as long as the ligula (Fig. 3C), which is 4.9 times as long in *L. miyamotoi* (Yoshitomi 2014: fig. 4F), and by the shape of antennomere 3 (Fig. 2A–C), which is about 1.1 times as long as wide (length and width about equal in *L. miyamotoi*; Nakane 1955: fig. 1).

##### Description.

Length 3.9–4.4 mm.

**Male**. Body black, head and pronotum with metallic greenish lustre; elytra with metallic bluish lustre; antennae with basal three segments orange; base of scape dark. Vestiture of dense, short pubescence.

Head with dorsal surface densely, finely punctured; frons with short, longitudinal groove; genae slightly constricted and smoother; frons covered with silver setae, longer in front of eyes. Eyes moderate in size and finely faceted, laterally protruding, with interfacetal setae. Antennae laterally inserted on frons, with insertions exposed from above. Antennae 11-segmented; antennomeres 4–10 semi-moniliform; antennomere 11 fusiform; scape elongated, subtriangular, with outer edge curved at base; antennomere 3 dilated and subtrapezoid anddorsal surface with small, rounded subapical depression and large concavity at base, bearing large membranous appendage on inner edge; anterior edge of basal concavity distinctly raised.

Pronotum about 0.8 times as long as wide; lateral margins slightly curved, with complete lateral carina; disc densely, finely punctured. Prosternum short; procoxal cavities large, transverse, contiguous at middle, widely opening to posterior. Scutellum transversely trapezoid, with posterior margin nearly truncate.

Elytra about 1.5 times as long as wide; lateral margins nearly parallel-sided; apex roundly detached; surfaces smooth, without distinct punctation; epipleura not extend to posterior margin of metaventrite. Metaventrite slightly dilated; metanepisternum broad. Procoxae and mesocoxae enlarged, protruding; trochanters large, subtriangular. Protibiae strongly enlarged and curved at base, with large concavity on inner edge, forming interlock structure with inner edge of profemora; meso- and metafemora slightly dilated; tibiae slender; tarsomeres 1–4 short and subequal in length; tarsomere 5 longest.

Abdomen with six freely articulated ventrites, nearly membranous; ventrite 1 divided at middle; last ventrite subtriangular. Tergite VIII subtrapezoid, anterolaterally with pair of short struts; posterior margin emarginate, covered with long, sparse setae along lateral and posterior edge; sternites VIII transverse, nearly divided at middle, covered with sparse, long setae along posterior edge. Specular gastrum sheath-shaped, broad. Aedeagus with penis constricted at middle, with apex slightly constricted and apical margin emarginate; endophallus with slender gonoporal piece and strongly curved, short ligula.

**Female**. Similar to male in shape and colouration, but with basal three antennomeres darker; scape and antennomere 3 only slightly dilated and elongated; profemora and protibiae simple, without concavity or tubercle along inner edges.

##### Etymology.

The species name refers to the Siming District of Xiamen, where the type series were collected.

##### Distribution.

Fujian (Xiamen, Quanzhou).

#### Laius xiamenensis sp. nov.

Taxon classificationAnimaliaColeopteraMelyridae

A4BCEC1A-32C7-5A1D-9C59-556ED734F88A

https://zoobank.org/68D219AF-FBE4-480D-BD1B-4F2326E093DF

Figs 1D–F, 2D–F, 2H, 2J, 3F–J, 4B

##### Chinese common name.

厦门滨囊花萤.

##### Type material.

***Holotype***: • male, “CHINA. Fujian, Xiamen, Siming District, Zhenzhuwan beach, E118.112624 N 24.429981, 2025.vi.7, leg. Zhou Qing” (IZGAS). ***Paratypes***: • 1 female same data with the holotype (IZGAS); • 7 males, 14 females, “CHINA. Fujian, Xiamen, Siming District, Zhenzhuwan beach, E118.112624 N 24.429981, 2025.vi.221–21, leg. Zhenhua Liu” (IZGAS); • 2 females, “CHINA Fujian, Xiamen, Siming District, Guanyinshan beach, E118.197125 N24.492729, 2025.vi.221–21, leg. Zhenhua Liu” (IZGAS); • 2 females, “CHINA, Fujian, Quanzhou, Jinmen County, E118.338654 N24.404996, 2025.vi.21, leg. Yuchen Zheng” (IZGAS).

##### Diagnosis.

This species belongs to species group 2 of Yoshitomi (2014) based on having the sclerites in the endophallus and the gonoporal piece with a basal projection (Fig. 3H). It resembles *L. flavicornis*, *L. lutaoensis*, and *L. zappii* Plonski, 2020 in colouration, the shape of the basal antennomeres and the following characters: scape long, strongly curved, and with tuft of long setae on outer side of apex, and antennomere 3 transverse and dorsal side with large excavation near inner edge (Yoshitomi 2008; Yoshitomi and Lee 2010; Plonski 2020). It can be separated from those species by the straighter ligula in the endophallus (Fig. 3H) and by the more acute outer margin of antennomere 3 (Fig. 2D, F).

##### Description.

Length 4.1–4.8 mm.

**Male**. Body black, with metallic bluish lustre; antennomeres 1–3, anterior edges of genae, tarsi, and basal parts of mouthparts orange; antennomere 4 orange to brownish. Vestiture of dense, short pubescence.

Head with dorsal surface densely, finely punctured; frons with small medial pit; genae moderately constricted; frons covered with silver setae, denser and longer on genae in front of eyes. Eyes moderate in size, finely faceted, laterally protruding, with interfacetal setae. Antennae laterally inserted on frons, with insertions exposed from above. Antennae 11-segmented; antennomeres 4–11 filiform; antennomere 11 fusiform; scape elongated, laterally flattened and twisted, with apex thickened, bearing tuft of golden setae; antennomere 3 dilated, transverse, with outer margin narrowly rounded, inner margin arcuate, dorsal surface with smaller elliptical depression near apex, and larger, deep concavity at base near inner edge, laterally bearing large membranous appendage, and anterior and inner edges of basal concavity distinctly raised.

Pronotum about 0.7 times as long as wide; lateral margins slightly arcuate, with complete lateral carina; disc densely, finely punctured. Prosternum short; procoxal cavities large and transverse, contiguous at middle, widely opening to posterior. Scutellum transversely trapezoid, with posterior margin nearly truncate.

Elytra about 1.5 times as long as wide, with lateral margins nearly parallel-sided, widest at about apical fourth, apex roundly detached; surfaces smooth, without distinct punctation; epipleura not extending to posterior margin of metaventrite. Metaventrite slightly dilated; metanepisternum broad. Procoxae and mesocoxae enlarged and protruding; trochanters large and subtriangular. Protibiae strongly enlarged and moderately curved at base, with large, longitudinal concavity on inner edge, forming interlock structure with inner edge of profemora; meso- and metafemora slightly dilated, tibiae slender; tarsomeres 1–4 short and subequal in length; tarsomere 5 longest; protarsi shorter than others.

Abdomen with six freely articulated ventrites, nearly membranous; ventrite 1 divided at middle, last ventrite subtriangular. Tergite VIII subtrapezoid, with pair of short struts anterolaterally, posterior margin emarginate, covered with long, sparse setae along lateral and posterior edges; sternites VIII transverse, nearly divided at middle, coved with long, sparse setae along posterior edge. Specular gastrum sheath-shaped. Aedeagus with penis slightly constricted near base; apex slightly constricted; apical margin emarginate; endophallus with slender gonoporal piece and slightly curved ligula; gonoporal piece with small basal projection.

**Female**. Similar in shape and colouration to male, but with scape and antennomere 3 only slightly dilated and elongated; front femora and tibiae simple, without concavity or tubercle on inner edges.

##### Etymology.

Species name refers to the type locality of the new species, Xiamen, a city of Fujian province.

##### Distribution.

Fujian (Xiamen, Quanzhou).

### Key to species of the genus *Laius* in China

(Male only; *L. pici* Miwa, 1931 and *L. theresae* Pic, 1944 are excluded)

**Table:** 

1	Antenna with scape strongly curved, apex with tuft of long setae	**2**
–	Antenna with scape subtriangular, apex without tuft of long setae	**4**
2	Antennomere 3 with outer edge narrowly rounded; ligula in endophallus distinctly curved	**3**
–	Antennomere 3 with outer edge broadly rounded; ligula in endophallus slightly curved	***L. xiamenensis* sp. nov**.
3	Antennomere 3 with inner edge broadly rounded; gonoporal piece in endophallus with small projection near base	***L. flavicornis***
–	Antennomere 3 with inner edge more narrowly rounded; gonoporal piece in endophallus with small projection at base	***L. lutaoensis***
4	Scape elongate; antennomere 3 subtrapezoid; endophallus with two sclerites	***L. simingensis* sp. nov**.
–	Scape stout; antennoemre 3 subquadrate; endophallus with three sclerites	***L. taiwanus***

## Discussion

The sympatric occurrence of the two newly described species is consistent with distribution patterns observed in many other *Laius* species (Yoshitomi 2014), when two or three species frequently co-occur the same locality. This phenomenon is also common within the tribe Apalochrini (personal observations from the first author).

**Figure 5.**
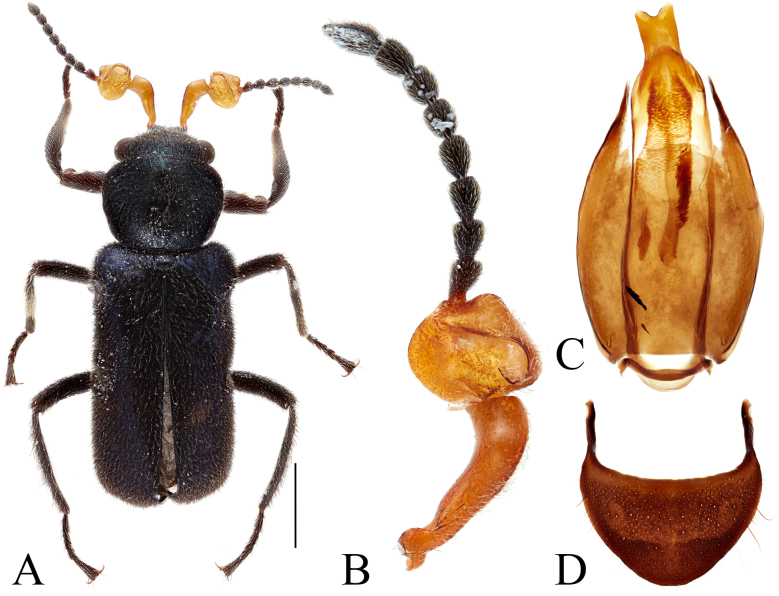
*Laius purpureipennis* Lea, 1916. **A**. Habitus, dorsal view; **B**. Antenna, dorsal view; **C**. Aedeagus, dorsal view; **D**. Tergite VIII. Scale bar: 1 mm.

*Laius* species typically exhibit narrow distribution ranges (Yoshitomi 2014). Given the extensive coastlines of eastern and southern China and the numerous islands in this region, further species are likely to be discovered. The record of *L. flavicornis* from China by Champion (1921) should be considered questionable for several reasons. It was originally described from Indonesia, the occurrence of this species in China does not conform to the narrow distribution pattern of species within the genus. Champion (1921) suggested that a specimen from Darwin, Australia, might also belong to *L. flavicornis*. However, a species from that region with similar modified male antennomeres was described as *L. purpureipennis* Lea, 1916 (Fig. 5A, B), which can be easily distinguished from *L. flavicornis* by the sclerites in the endophallus and the shape of tergite VIII (Fig. 5C, D). Similarly, *L. xiamenensis* shares similar colouration and basal antennomere morphology with *L. flavicornis* but possesses a distinctly a different aedeagus. This problem cannot be solved until examination of the specimens preserved in The Natural History Museum, London.

## Supplementary Material

XML Treatment for Laius

XML Treatment for Laius simingensis

XML Treatment for Laius xiamenensis

## Additional information

### Artificial Intelligence (AI) use

The authors accept full responsibility for the content of the manuscript, including the disclosure of any use of AI.

We used Qwen developed by Alibaba to check the English grammar and writing of this manuscript.

### Author ORCIDs

Z. Liu https://orcid.org/0000-0002-2739-3305

F. Qiu https://orcid.org/0000-0003-3552-7147

Z. Li https://orcid.org/0000-0003-0064-7165

### Data availability

All of the data that support the findings of this study are available in the main text.
